# Design and Mechanism of Action of a New Prototype of Combi-Molecule “Programed” to Release Bioactive Species at a pH Range Akin to That of the Tumor Microenvironment

**DOI:** 10.3390/ph14020160

**Published:** 2021-02-16

**Authors:** Anne-Laure Larroque-Lombard, Etienne Chatelut, Jean-Pierre Delord, Diane-Charlotte Imbs, Philippe Rochaix, Bertrand Jean-Claude, Ben Allal

**Affiliations:** 1 Centre de Recherches en Cancérologie de Toulouse (CRCT), Institut Claudius-Regaud–Institut Universitaire du Cancer Toulouse-Oncopole and UMR 1037 INSERM, 31052 Toulouse, France; anne-laure.larroque@mail.mcgill.ca (A.-L.L.-L.); chatelut.etienne@iuct-oncopole.fr (E.C.); Delord.Jean-Pierre@iuct-oncopole.fr (J.-P.D.); Imbs.DianeCharlotte@iuct-oncopole.fr (D.-C.I.); Rochaix.Philippe@iuct-oncopole.fr (P.R.); 2 McGill University Health Center (RI-MUHC), 1001 Decarie Blvd, Research Institute, Montreal, QC H4A 3J1, Canada

**Keywords:** combi-molecules, MEK, synergy, microenvironment, pharmacokinetics

## Abstract

The clinical use of cytotoxic agents is plagued by systemic toxicity. We report a novel approach that seeks to design a “combi-molecule” to behave as an alkylating agent on its own and to undergo acid-catalyzed conversion to two bioactive species at a pH range akin to that of a tumor microenvironment: an AL530 prototype was synthesized and we studied its ability to release a chlorambucil analogue (CBL-A) plus a potent mitogen-activated protein/extracellular signal-regulated kinase kinase (MEK) inhibitor (PD98059) at different pHs in buffered solutions, plasma and tumors. Its potency was compared in vitro with CBL+PD98059 (SRB assay) and in vivo in a xenograft model. Its target modulation was studied by western blotting and immunohistochemistry. AL530 released PD98059+CBL-A at mild acidic pH and in vitro was fivefold more potent than CBL and three-to-fivefold more potent than CBL+PD98059. In vivo it released high levels of PD98059 in tumors with a tumor/plasma ratio of five. It induced γ-H2AX phosphorylation and blocked pErk1,2, indirectly indicating its ability to damage DNA and modulate MEK. It induced significant tumor delay and less toxicity at unachievable doses for CBL and CBL+PD98059. We demonstrated the feasibility of a pH-labile combi-molecule capable of delivering high MEK inhibitor concentration in tumors, damaging DNA therein, and inducing tumor growth delay.

## 1. Introduction

**Figure 1:**
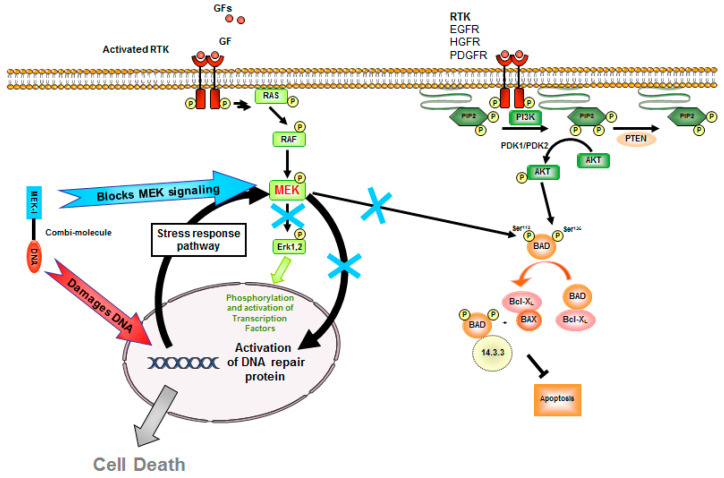
Multiple cell signaling pathways leading to MEK activation. GF: Growth Factor, RTK: Receptor Tyrosine Kinase.

In advanced stages, the clinical management of solid tumors is primarily based on the combination of potent cytotoxic agents. The therapeutic activity of these regimens is often hampered by severe toxicity and has acquired resistance in refractory tumors. In order to improve the therapeutic index, efforts are now directed at combinations of cytotoxic agents with targeted therapy. In this context, over the past decade, we developed a novel approach termed “combi-targeting” that seeks to design molecules termed “combi-molecules” to block receptor tyrosine kinase-mediated signaling while carrying a cytotoxic DNA-damaging moiety [1,2,3,4,5,6,7]. The fundamental premise of the approach was to bring together multiple mechanisms of action in a single molecule, thereby reducing the pharmacotoxicology associated with multiple drug-related mechanisms to that of a single agent. This approach will thus reduce the incidence of additive toxicity associated with classical combinations. Three types of combi-molecules (type I, II and III) emerged [8]. As depicted in Figure 1, type I combi-molecules contain a moiety targeted to oncogenic kinase and another to DNA. Type I combi-molecules can inhibit the function of their target kinase while remaining intact. However, hydrolysis is required for the generation of the cytotoxic DNA-damaging species. Type II combi-molecules are chimeric structures that do not require hydrolysis to elicit the dual-targeting activities. Recently, we reported on the first type III combi-molecules that were designed to hit their two targets while remaining intact, but also to slowly fragment into two smaller inhibitors of the latter targets [8]. The proof-of-concept of the three types of combi-molecules was performed using the epidermal growth factor receptor (EGFR) tyrosine kinase inhibitor combined with DNA dual-targeted agents such as RB24 (type I) [2,3,9] or ZR2003 (type II) [10,11] that showed extremely strong growth inhibitory potency against cells overexpressing EGFR. AL776, the first type III combi-molecule synthesized, targeted EGFR and Src, two oncogenic tyrosine kinases that synergize to promote growth and invasion [8]. However, despite encouraging results for the three types of combi-molecules, advancement toward clinical trials have been hampered by the instability of the type I molecules in plasma, while type II are still under development toward clinical trials [12]. The increased molecular size of type III combi-molecules is deleterious to absorption in vivo. To circumvent these problems, we proposed an alternative approach that consists of designing type I combi-molecules to be hydrolyzed under tumor “microenvironment” conditions. This strategy was designed to allow the molecule, regardless of its size, to be cleaved at the tumor site, generating small and diffusible species with distinct mechanisms of action. Cell penetration is thus improved while preserving the target multiplicity of the combi-targeting principle.

The strategy to target MEK and DNA in this combi-molecule was inspired by common knowledge that tumor microenvironment and stromal tissues drive the growth of solid tumors, predominantly through the secretion of growth factors. When tumors overexpress receptors responding to these growth factors, their progression is accelerated by both uncontrolled proliferation and invasion. Signaling mediated by these receptors converges toward the activation of MEK, which is at the cross-roads of the Ras-mitogen-activated protein kinase (MAPK) and stress response pathways. Activation of many growth factor receptors [e.g., EGFR, hepatocyte growth factor receptor (HGFR) orMet, platelet-derived growth factor (PDGFR), fibroblast growth factor (FGFR)] leads to gene expression through the MAP kinase pathway in which the protein kinases MEK1,2 (henceforth referred to as MEK) play a major role (Figure 1). Along with other authors [1,13], we have shown that DNA damage (alkylation or radiation) triggers expression of the DNA repair proteins X-ray repair cross-complementation group 1 (XRCC1) and excision repair cross-complementation group 1 (ERCC1) through MEK activation via the stress response pathway. We also showed that concomitant DNA damage induction and growth stimulation by growth factors (e.g., EGFR) additively enhances the expression of the DNA repair protein XRCC1 [3]. Concomitant inhibition of growth factor-induced signaling and induction of DNA damage were found to induce synergistic cell-killing [3].

**Scheme 1:**
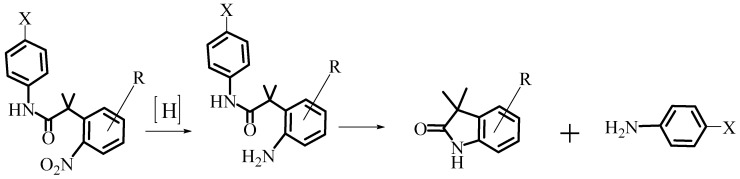
2-Nitroaryl amides proposed as bioreducible prodrugs of anilines based on the intramolecular cyclization of the 2-aminoaryl amides resulting from the reduction of the 2-nitro group.

The drug design approach described herein is also influenced by the hypoxic conditions of the tumor microenvironment. Indeed, in advanced stages, tumors contain hypoxic areas and undergo hypermetabolism that increases acid production, thereby lowering the pH of the microenvironment [14,15,16]. We therefore surmised that a strategy seeking to design a molecule capable of releasing an aniline mustard (CBL-A, Scheme 1) and a MEK inhibitor in the mildly acidic milieu of the tumor microenvironment could lead to molecules capable of inducing strong potency against refractory tumor. We report herein on the feasibility of this approach by designing AL530 containing a pH-sensitive o-amino-phenyldimethylacetamide linker and elucidate its mechanism of action in vitro and in vivo. The study was performed in cells overexpressing EGFR, a receptor acting primarily through the MAPK pathway. To our knowledge, this is the first report on such a type of molecule.

## 2. Results

### 2.1. Design and Synthesis of AL530

**Scheme 2:**
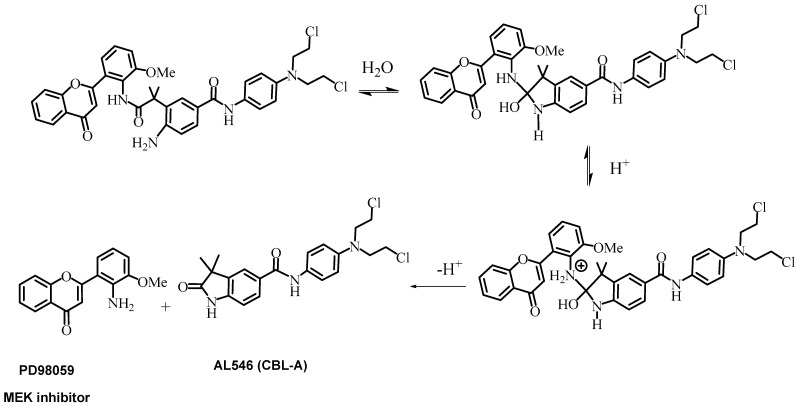
Proposed mechanism for the pH dependence of the degradation of the lead combi-molecule AL530.

**Scheme 3:**
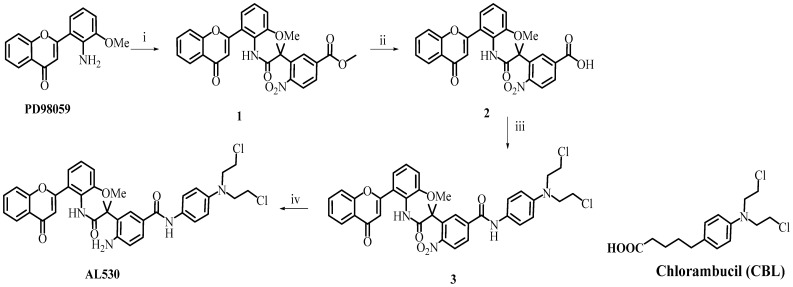
(**i**) **8S**, 1 h and THF/CH_2_Cl_2_ 1/1, Et_3_N, 0 °C to 50 °C, 3 days, 44%, (**ii**) LiOH 2N, MeOH, rt, 18 h, 97%, (**iii**) CDI, aniline mustard (compound 12S), THF/ACN 1/1, 0 °C and rt, 18 h, 47%, (**iv**) Pd/C 10%, MeOH, H_2_, rt, 3 h, 65%.

The approach we chose to study was to design a DNA-damaging aniline mustard with a masked MEK inhibitor. The latter was “programed” to be released under the mild acidity of the tumor microenvironment, thereby locally sensitizing tumors to the cytotoxic effects of the released CBL-A. Our molecular design was inspired by previous work reported by Atwell et al. [17] who, in an attempt to prepare bioreducible nitroaryl amide prodrugs, studied the intramolecular cyclization of o-aminoaryl amides and observed a pH dependence of the cleavage. As outlined in Scheme 1, cyclization of the o-aminoaryl amides led to benzimidazolones and free anilines. Thus, we designed AL530 to carry the aminoflavone as in Scheme 2 with the unique positioning of an amino group in close proximity to the amide linker carrying the PD98059 warhead. PD98059 (Scheme 2) is a potent allosteric MEK inhibitor that loses its activity upon substitution or repositioning of its amino group on the flavone ring [18,19]. Briefly, as depicted in Scheme 2, an acid-catalyzed intramolecular addition of the o-amino group onto the amide carbonyl would lead to the release of intact PD98059. The synthesis of AL530 is shown in Scheme 3. Details of the synthesis of the key synthons are given in the Supporting Information section. The synthesis of PD98059, the acid chloride **8S** and the aniline mustard **12S** are outlined in Schemes S1–S3 (see supporting information), respectively, as per previously published methods. In Scheme S1, to facilitate the understanding of the conversion of 1S to 2S, arrows are used to indicate that it results from an intramolecular reaction between the base-activated alpha ketone and the carbonyl of the phenolic ester. As depicted in Scheme 2, AL530 was synthesized in four steps. In summary, intact PD98059 was treated with **8S** under basic conditions to produce **1**, which was deprotected under basic conditions to give acid **2**. Coupling of acid **2** with the 4-amino aniline mustard **12S** gave **3**, the nitro group of which was catalytically reduced to give AL530 in good yield. Various observations showed that AL530 to be acid- and temperature-sensitive. Therefore, we sought to verify whether its hydrolysis could occur at a pH range akin to that of the tumor microenvironment. To ascertain the kinetics of its stability under acidic and variable temperatures, a rigorous pH and variable temperature study was performed as follows.

### 2.2. Kinetics of AL530 Hydrolysis at Variable pH and Temperature

**Figure 2:**
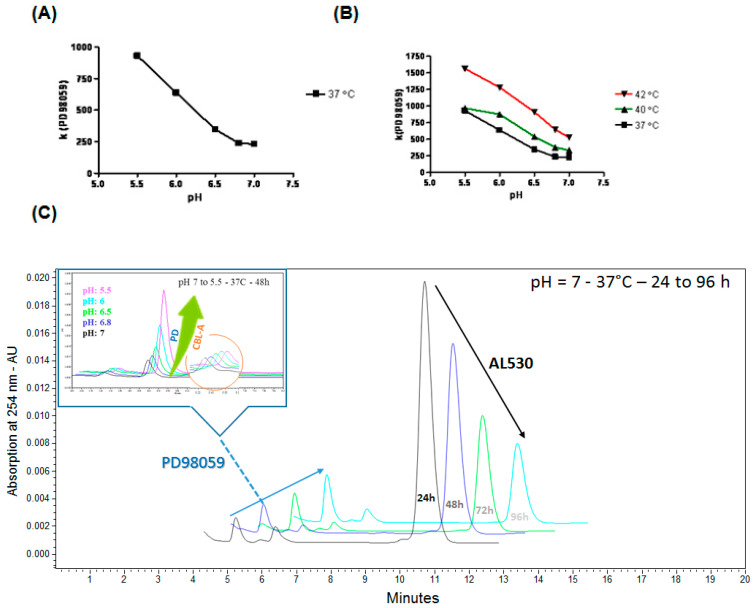
Influence of pH and temperature on AL530 hydrolysis. Graphs (**A**) and (**B**) represent the formation rate (constant k) of PD98059 at ■—37 °C, ▲—40 °C and ▼—42 °C versus the pH ranging from 5.5 to 7. The overlaid chromatograms (**C**) represent the time course effects (from 24 to 96 h) of AL530 hydrolysis versus the formation of PD98059 at 37 °C at pH = 7.

To study the pH and temperature influence on the kinetics of AL530 hydrolysis into its two drug components, we used a LC–UV instrument to determine the rate of formation (k) of PD98059 under these different conditions: pH ranging from 5.7 to 7 and temperatures of 37 to 42 °C. As shown in Figure 2, the conversion of the combi-molecule AL530 into its two bioactive components (DNA damage species and MEK inhibitor) was largely accelerated at an acidic pH. The pH rate profile also indicated that AL530 is hydrolyzed at a pH range between 5.5–6.5, which is within the range of pH reported for the tumor microenvironment [12,13,14]. The results are in line with the cleavage mechanism proposed in Scheme 1, wherein the pH rate profile clearly suggests that N-protonation of the tetrahedral intermediate is the rate determining step of the reaction.

As depicted in Figure 2B, the rate of hydrolysis is not only pH-dependent but also accelerated with increasing temperatures. Minor temperature changes (e.g., 37–42 °C) led to a dramatic acceleration of the rate of conversion of AL530 to PD98059. This may well be due to the close proximity of the amino group to the carbonyl of the linker.

The interest in using higher temperatures than physiological conditions (37 °C) was driven by various housekeeping works and medical care in our institution using hyperthermic intraperitoneal chemotherapies [20,21]. Indeed, we and others have showed dramatic potentiation of the cytotoxic effect of certain chemotherapy agents with enhancement of the tissue penetration of the administered drug at 42 °C compare to 37 °C. As shown in a chromatograms illustration (Figure 2C), the conversion of the combi-molecule AL530 into its two bioactive components (DNA damage species-mustard: CBL-A and MEK inhibitor: PD98059) was increased in a time–environment acidification and temperature-dependent manner.

### 2.3. Growth Inhibition of AL530

In order to establish that AL530 can inhibit cell growth, a panel of solid tumor cell lines of different origins (head and neck cell lines: HN, CAL166 and CAL33; prostate: DU145; breast: MDA-MB -468; lung: A427, Table 1) were exposed to AL530 and the results were compared with those obtained from the same panel exposed to CBL, a clinical aniline mustard and intact PD98059 (Table 2). The cells were exposed to different doses of AL530, CBL, PD98059 and combinations of CBL plus PD98059 for four days to maximize the probability of PD98059 release. As shown in Table 2, AL530 induced strong growth inhibitory potency with IC50 values in the low micromolar range despite the neutral pH of the cell culture medium. Its potency was consistently three-to-fivefold stronger than that of CBL. More importantly, AL530 showed two-to-threefold stronger activity when compared with the combination of CBL+PD98059, indicating that its mechanism of action may be different from that of a simple two-drug combination of an MEK inhibitor with an aniline mustard.

### 2.4. Erk Phosphorylation Inhibition by AL530

**Figure 3:**
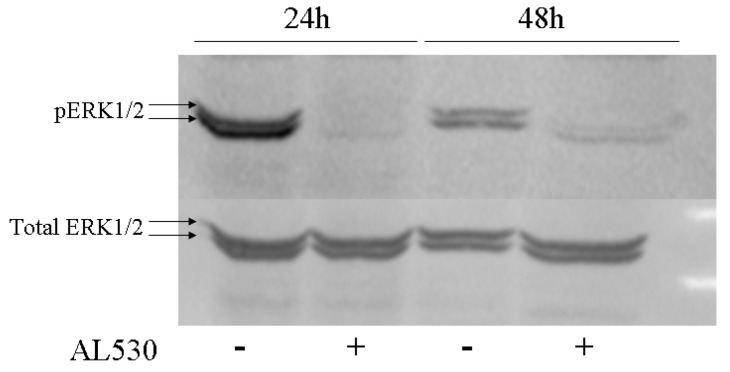
Study of Erk1,2 phosphorylation inhibition in CAL33 cells daily treated with AL530 (25 µM) for 24 to 48 h. At the indicated times in the figure, cells were harvested, lysed (RIPA buffer), an equal amount of protein was separated (12.5% SDS PAGE gel), blotted (PVDF membrane) and coated with antibody of interest.

As mentioned earlier, MEK is activated through the Ras-MAP kinase pathway in response to receptor-mediated and stress-response signaling. In order to determine whether AL530 was capable of inhibiting MEK and its Erk1,2 substrates in cells, we examined its effect on the phosphorylation status of MEK. Given that under the treatment conditions, the combi-molecule can only be activated in the intracellular milieu, the assay was performed with long exposure times (24–48 h) and at a relatively high concentration of AL530 (25 µM). This 25 µM dose was selected to maximize the probability of releasing free PD98059. As shown in Figure 3, the study of the Erk1,2 phosphorylation level in CAL33 cells exposed to 25 µM of AL530 for 24 and 48 h showed a dramatic inhibition of Erk1,2 phosphorylation.

### 2.5. Pharmacokinetics and Pharmacodynamics of AL530

The observed in vitro cellular and molecular mechanisms of action of AL530 strongly suggest that it possesses all the characteristics required to become the lead prototype of the approach under study. While these characteristics were evidenced in vitro, the pharmacokinetics and pharmacodynamics study of AL530 in vivo remained the ultimate test for the feasibility of the approach. Prior to starting a human xenograft model, a preliminary in vivo biodistribution study was conducted in the 4T1 breast syngeneic mouse model that allows to form large tumors in vivo in a very short time. In a second instance, we selected from the panel of cancer cell lines of the study, the human CAL33 head and neck cancer cells to perform a xenograft model, on the basis of its aggressive growth in vivo.

#### 2.5.1. Study of the Release of PD98059 and CBL-A in Plasma and Tumor

**Figure 4:**
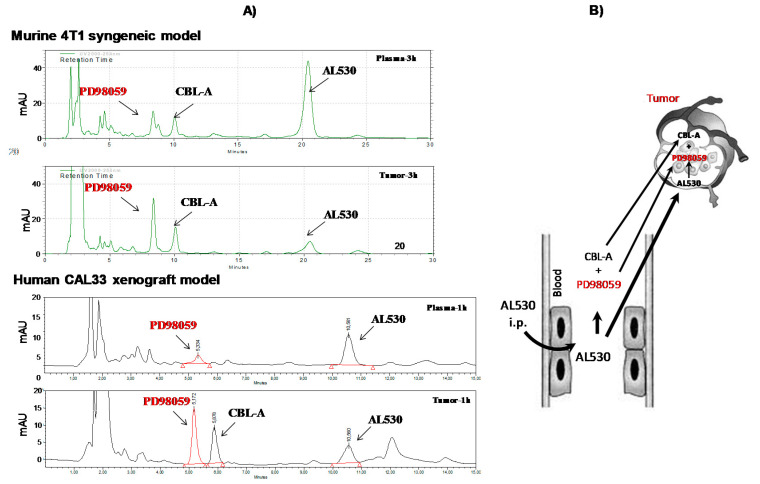
(**A**) In vivo hydrolysis of AL530 in 4T1 mouse mammary tumors (top panel) and human head and neck tumors generated from the CAL33 human xenograft in nude mice (bottom panel). Each chromatogram represents the analysis of plasma or tumor extract from a mouse treated with 100 mg/kg of AL530 (i.p.) after 3 h (4T1 model) and 50 mg/kg (i.p.) after 1 h (CAL33 model). Following mice sacrifice, plasma and tissue were collected and stored at −80 °C until the day of analysis by HPLC as described in the Materials and Methods section. Consistently higher levels of PD98059 were released in tumor tissues than in plasma. (**B**) Proposed model for the absorption and distribution of AL530 and the localization of its hydrolytic conversion to PD98059 + CBL-A.

4T1 cell (murine)-bearing mice were given AL530 i.p., and plasma and tumors were collected after 3 h and analyzed by HPLC. As depicted in Figure 4, the results showed remarkably higher levels of PD98059 in tumors than in plasma. These data stimulated our interest in carrying out an in vivo study in a more clinically relevant human head and neck cancer cell xenograft. Accordingly, the experiments were repeated with CAL33-implanted mice after 1h of exposure to AL530 to show a markedly more abundant release of PD98059 in the tumors when compared with plasma in these mice.

To further ascertain these results, a full pharmacokinetics study involving analyses at various time points was performed with CAL33-bearing mice. The results, which are summarized in Table 3, showed that using the AUC_0–24h_-PD98059 (tumor)/AUC_0–24h_ (plasma) ratio was consistent with an approximately fivefold more abundant release of PD98059 from AL530 in the tumors than in the plasma. Overall the tumor/plasma ratio of intact AL530 was 1.6 and that for PD98059, the ratio was 5, which is again consistent with a preferential tumor distribution of AL530 and its derived primary metabolite PD98059.

#### 2.5.2. Pharmacodynamics/Immunohistochemistry Analysis of AL530 Effect on Tumor Tissue

**Figure 5:**
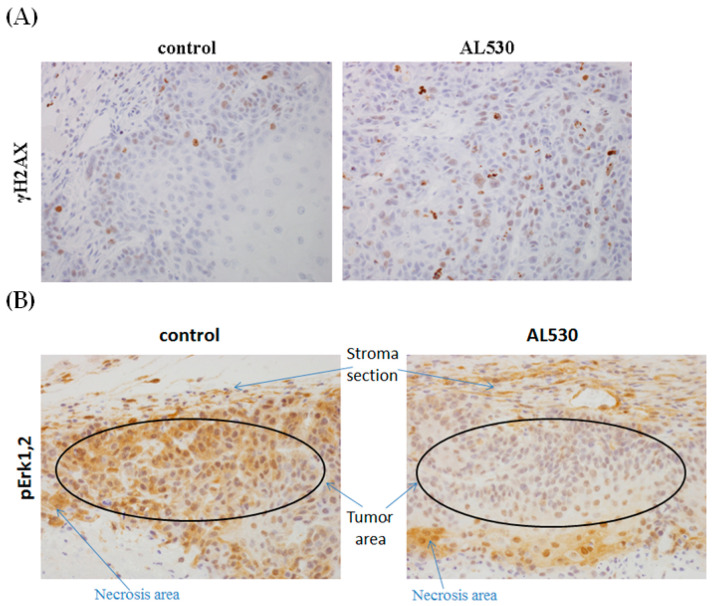
Immunohistochemistry analysis of the effect of AL530 on tumor tissue. Thick sections of 4 µm formalin fixed and paraffin embedded in tumors from CAL33 cell xenografts in nude mice treated with vehicle or 50 mg/kg of AL530 i.p. for 13 days, were stained with antibody against γ-H2AX (**A**) and pErk1,2 (**B**) (magnification, x20).

In vitro assays showed that AL530 was capable of blocking MEK after a long exposure time. Given the preferential tumor distribution of AL530 and the higher concentration of PD98059 within tumors, we analyzed its ability to modulate its two targets in vivo: DNA for CBL-A or intact AL530 and MEK for PD98059. This was studied using γ-H2AX (Figure 5A) immunostaining to indirectly determine its ability to damage DNA and pErk1,2 to assess its ability to block MEK (Figure 5B) in vivo. Our results showed that, indeed, AL530 dramatically inhibited Erk1,2 phosphorylation (Figure 5B). Conversely, increased γ-H2AX histone staining was observed, which indirectly confirms its ability to damage DNA in these tumors (Figure 5A).

#### 2.5.3. Efficacy of AL530 in Vivo

##### Dose Tolerance Efficacy

**Figure 6:**
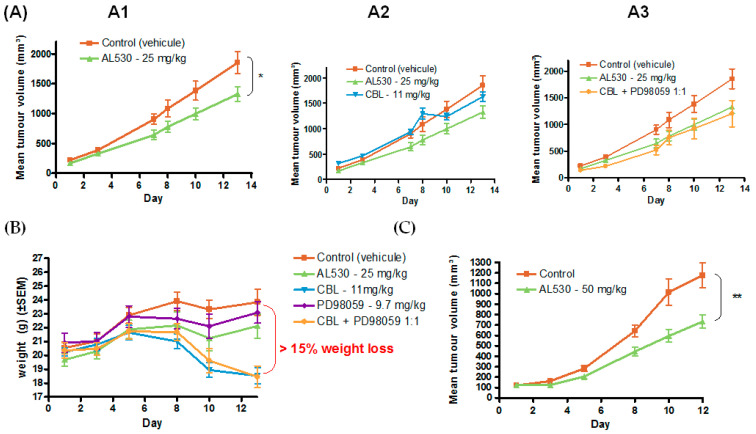
Effect of vehicle, AL530, Chlorambucil (CBL), PD98059 (MEK inhibitor) or equimolar combination CBL + PD98059 treatment on tumors growth (**A**,**C**) and weight (**B**) properties of CAL33 head and neck cancer cell xenograft. Nude mice (*n* = 7 per group) bearing subcutaneous 200 mm^3^ (at the start of the experiment) bilateral CAL33 tumors xenograft received either saline (orange square, **A1**), 25 mg/kg (**A1**) or 50 mg/kg (**C**) of AL530 (green triangle), or 11 mg/kg of CBL (**A2**, blue diamond), or 9.7 mg/kg of PD98059 (data not shown) or an equimolar combination of CBL + PD98059 (1:1; **A3**, orange circle) daily by i.p. route/q5d throughout the experiment. Data are expressed as mean ± SEM. ** *p* < 0.0001 and * *p* < 0.01, Mann–Whitney posthoc test.

The dose tolerance study of AL530 was based on known values for CBL, used as a positive control. While CBL showed signs of acute toxicity at 11 mg/kg both as a single drug or in combination with PD98059, AL530 could be tolerated at 25 and 50 mg/kg (Figure 6). Higher doses (e.g., 100 mg/kg) were not achievable due to the poor solubility of the drug at these doses in the vehicle. These results showed that AL530 is significantly less toxic than CBL.

The in vivo efficacy (Figure 6A,C) and toxicity (B) of AL530 were studied in nude mice bearing CAL33 head and neck cancer cells. Mice were treated with AL530, CBL, PD98059 (MEK inhibitor) or an equimolar combination of CBL with PD98059. PD98059 treatment is not represented in the graph because it was virtually inactive alone (data not shown). AL530 was administered at 25 mg/kg and 50 mg/kg (a dose at which CBL is toxic). Significant antitumor activity was observed for the AL530 combi-molecule at 25, and 50 mg/kg (*p* < 0.01 and *p* < 0.0001, respectively). By contrast, combinations of CBL with PD98059 or CBL alone did not show significant tumor delay in the model. Strikingly, AL530 showed a better tolerance in vivo than CBL and its combination with PD98059. The combi-molecule was tolerated at 25 and 50 mg/kg, two doses that were not achievable by CBL alone or in combination. At doses as low as 11 mg/kg, more than 15% weight loss was observed in the animals 13 days after treatment (B).

## 3. Discussion

Over the past two decades, resistance to DNA alkylating drugs has been primarily associated with DNA repair enzyme levels and activity. Advances in molecular biology led to the elucidation of the role of upstream cell signaling in the modulation of DNA repair activity. This progress inspired therapeutic interventions directed at receptor- or stress-mediated signaling to sensitize refractory tumors to DNA interactive drugs and radiation. In previous work, we and others [2,22], showed that blocking MEK, a signaling protein at the cross-roads of receptor- and stress-induced signaling, sensitized cells to alkylating agents and radiation. However, despite the ample evidence of synergy between the latter events (DNA damage and cell signaling inhibition), strategies to enhance the tumor selectivity of the resulting combinations are still lacking. Our results provide prima facie evidence of a novel strategy to enhance tumor selectivity by enabling a single molecule to activate mechanistic events triggered by the conditions of the tumor microenvironment. Here, the single molecule AL530 was designed to preferentially trigger the synergistic events (i.e., the inhibition of MEK and the induction of DNA damage) under the mild acidic pH of the tumor microenvironment.

AL530 is a combi-molecule with three compartments: (a) one masking the release of the kinase inhibitor, (b) another containing the anchimeric assistance engine designed to release the masked inhibitor under controlled conditions and (c) an aniline mustard carrier that is a precursor of another molecule (CBL-A) targeted to DNA. Thus, AL530 is a novel, first-in-class aniline mustard “programed” to generate an inhibitor of MEK, or more simply an aniline mustard designed to be a MEK inhibitor strictly under hydrolytic conditions favored by the tumor microenvironment. Following its successful design and synthesis, we sought to demonstrate first that it was indeed a prodrug of PD98059.

Our results unequivocally demonstrated that AL530 was capable of generating PD98059 plus a mustard-carrying metabolite in a pH- and temperature-dependent fashion. The pH rate profile suggests that protonation of the tetrahedral intermediate can be proposed at the rate determining step of the cleavage process. The results show that AL530 behaves as a prodrug of PD98059 at physiological pH and temperature ranges. Indeed, previous reports confirmed using specific probes that the pH of the microenvironment could be as low as 5.5 [14,15,16].

The study of the temperature-dependent cleavage of AL530 was performed in vitro. The purpose of the evaluation of temperature superior to 37 °C (40 and 42 °C) was to investigate whether this molecule could also be used for thermotherapy. Currently, hyperthermic intraperitoneal chemotherapies (HIPECs) are used in the clinical management of ovarian cancer. It consists of directly delivering highly concentrated and stably heated platinum-based chemotherapy treatments at 42 °C to the abdomen of the patient [20,21]. As shown in the pH study, the rate of AL530 cleavage was significantly accelerated in the 40 °C temperature range. The observed data suggest that the thermolability of AL530 may be an additional property that could be exploited, in addition to its pH lability, to kill tumor cells locally.

AL530, being an aniline mustard, means that its growth inhibitory potency was compared to that of the clinical analogue CBL under standard conditions in a monolayer culture. Moreover, continuous exposure studies showed AL530 to be fivefold more potent than CBL as well as two-to-threefold more potent than equimolar combinations of CBL + PD98059. This indicated that perhaps AL530 exerted its antitumor activities by a mechanism different from that of CBL alone. Given that, in the cell culture medium, the rate of hydrolysis of AL530 was found to be significantly slower (data not shown), the molecule probably owes its superior activity to a faster cleavage in the intracellular milieu. This concurs with the dissection of its mechanism of action that showed its ability to block Erk1,2 phosphorylation in CAL33 cells under a four-day continuous exposure assay. These observations were prima facie evidence that the hydrolysis of AL530 was milieu-dependent, thus warranting further investigation in vivo.

Given the dependence of AL530 cleavage on pH, plasma and tumor pharmacokinetics were the most appropriate models for studying its mechanism of action. The first striking observation was that the pharmacological half-life for AL530 was twice as long in tumors than in plasma. Interestingly, it was four times longer for PD98059 in tumor tissues, suggesting that the inhibition of MEK could be sustained even after AL530 has been cleared from the tumors. All pharmacokinetic parameters were remarkably consistent with a preferential distribution of AL530 and PD98059 in the tumors. The areas under the curves (AUCs), which represent exposure at all time points showed a tumor/plasma ratio, respectively, of 1.6 for AL530 and of 5 for PD98059. Since this was consistent with strong tumor distribution, we dissected the mechanism of action of AL530 in immunocompromised mice 13 days after drug administration. High staining for γ-H2AX suggested the presence of DNA damage, and decreased staining for pErk1,2 indicated the inhibition of phosphorylation in vivo. Taken together, the results were consistent with the in vitro observation and further support the feasibility of developing a molecule capable of massively accumulating in the tumor tissue and releasing its bioactive kinase inhibitor therein, despite its large size (MW = 6872 g/mol).

One of the premises of the combi-targeting concept is that by imprinting multiple mechanisms of action within a single molecule, the pharmacotoxicology of multiple mechanisms associated with multiple drugs will be reduced to that of a single agent. This postulate is supported by the results obtained herein, which showed AL530 to be much better tolerated than CBL alone or in combination with PD98059. A maximum tolerated dose could not be defined due to the insolubility of the drug at high doses. Nevertheless, significant tumor delay was observed at 25 and 50 mg/kg, indicating that further strategies for enhancing solubility may lead to the full-blown potency of the approach in vivo.

## 4. Materials and Methods

### 4.1. Chemistry

^1^H NMR spectra were recorded on a Varian 300 or 400 MHz spectrometer. Chemical shifts are given as δ values in parts per million (ppm) and are referenced to the residual solvent proton peak. Mass spectrometry was also performed on a Finnigan LC QDUO spectrometer by the McGill University Mass Spectroscopy Center using electrospray ionization (ESI) mode. Data are reported as m/z (intensity relative to base peak = 100). All chemicals were purchased from Sigma–Aldrich (now Millipore Sigma Canada Co. Oakville, Ontario Canada) or Alfa Aesar (Tewksbury, MA, United States). The compound SL02548 was purchased from Sinova (Elkridge, Maryland, United States).

#### 4.1.1. Compound **1**

The 2-(5-(Methoxycarbonyl)-2-nitrophenyl)-2-methylpropanoic acid linker, compound 7S (3.857 g, 0.014 mol), was refluxed in neat SOCl_2_ (6 mL) for 1 h (compound 8S), the mixture was then evaporated and thoroughly vacuum-dried to obtain a brown solid. PD98059 (2.565 g, 1 eq.) was dissolved in dry CH_2_Cl_2_ (200 mL) with Et_3_N (4 mL, 3 eq.) at 0 °C, and the acyl chloride formed was dissolved in THF (130 mL) and added dropwise to the cold solution via a canula. The reaction mixture was heated at 50 °C under argon for three days. It was then evaporated and the resulting crude material was resuspended in CH_2_Cl_2_ and extracted with brine. The organic layer was dried over magnesium sulphate, filtered and evaporated. The orange crude product was purified by silica gel chromatography (CH_2_Cl_2_ 100% to CH_2_Cl_2_/MeOH 8/2) to give compound (Scheme 3) as a pure powder (3.257 g, 44%). ^1^H NMR (300 MHz, CDCl_3_ δ ppm) 1.25 (s, 3H), 1.74 (s, 3H), 3.87 (s, 3H), 3.97 (s, 3H), 6.39 (s, 1H), 6.70 (dd, *J* = 8.8, 2.5 Hz, 1H), 7.37 (m, 1H), 7.50 (m, 2H), 7.64 (m, 1H), 7.64 (m, 1H), 7.84 (d, *J* = 8.8 Hz, 1H), 7.95 (m, 1H), 8.06 (m, 1H), 8.22 (dd, *J* = 8.0, 1.7 Hz, 1H), 8.29 (d, *J* = 1.7 Hz, 1H).

#### 4.1.2. Compound **2**

The ester **1** (3.257 g, 0.0063 mol) was hydrolyzed in MeOH (50 mL) with an aqueous solution of LiOH 2N (25 mL) added dropwise. The mixture was stirred at room temperature during 18 h and the reaction mixture was then acidified with an aqueous solution of HCl 1N to pH = 2. The precipitate formed was extracted with ethyl acetate; the organic layer was washed with water and brine, dried over magnesium sulphate, filtered and evaporated. The yellow solid, not further purified, gave compound **2** (3.061 g, 97%).^1^H NMR (300 MHz, CDCl_3_ δ ppm) 1.24 (s, 3H), 1.67 (s, 3H), 3.76 (s, 3H), 6.89 (s, 1H), 7.02 (m, 1H), 7.29 (d, *J* = 4.7 Hz, 1H), 7.45 (m, 2H), 7.58 (d, *J* = 8.3 Hz, 1H), 7.72 (m, 1H), 7.98 (d, *J* = 8.3 Hz, 1H), 8.15 (d, *J* = 8.3 Hz, 1H), 8.27 (d, *J* = 8.3 Hz, 1H), 8.42 (s, 1H).

#### 4.1.3. Compound **3**

A stirred solution of compound **2** (336 mg, 0.668 mmol) in dry N,N-dimethylformamide(DMF)/acetonitrile (ACN) (3/4 mL) was treated with 1,l’-carbonyldiimidale (120 mg, 1.1 eq.) at room temperature for 30 min and then cooled to 0 °C and treated with a solution of aniline mustard **12S** (205 mg, 1 eq.) in ACN (7 mL) with Et_3_N (280 µL, 3 eq.). The reaction mixture was warmed to room temperature and stirred under argon. After 18 h, the DMF was azeotroped with heptane, and the dark oil obtained was resuspended in ethyl acetate and extracted with water. The organic layer was dried over magnesium sulfate, filtered and evaporated. The dark orange oil obtained was purified by silica gel chromatography (CH_2_Cl_2_/EtOAc 8/2 to 7/3) to give compound **3** in the form of a pure orange powder (225 mg, 47%). ^1^H NMR (300 MHz, CDCl_3_ δ ppm) 1.63 (s, 6H), 3.63 (m, 4H), 3.72 (m, 4H), 3.86 (s, 3H), 6.43 (s, 1H), 6.62 (d, *J* = 9 Hz, 2H), 7.04 (dd, *J* = 8.2, 1.6 Hz, 1H), 7.19 (m, 1H), 7.27 (m, 1H), 7.36 (m, 1H), 7.42 (d, *J* = 7.8 Hz, 1H), 7.51 (d, *J* = 9 Hz, 2H), 7.62 (m, 2H), 7.75 (d, *J* = 1.6 Hz, 1H), 7.78 (s, 1H), 8.08 (dd, *J* = 1.6 Hz, 1H), 8.12 (dd, *J* = 7.8, 1.6 Hz, 1H).

#### 4.1.4. Compound AL530

A solution of the nitro compound **3** (225 mg, 0.0003 mol) in MeOH (90 mL) was hydrogenated for 3 h over Pd-C 10% (450 mg) at room temperature. The solution was filtered through celite, and the solvent was evaporated. The crude material was purified by silica gel chromatography (CH_2_Cl_2_ /EtOAc 6/4 to 4/6) to give AL530 as a pure light yellow powder (140 mg, 65%). ^1^H NMR (400 MHz, DMSO-*d*_6_ δ ppm) 1.45 (s, 6H), 3.65 (br s, 8H), 3.75 (s, 3H), 5.16 (s, 2H), 6.44 (s, 1H), 6.65 (d, *J* = 8.6 Hz, 1H), 6.71 (d, *J* = 9 Hz, 2H), 7.24 (d, *J* = 8.2 Hz, 1H), 7.29 (d, *J* = 6.7 Hz, 1H), 7.43 (m, 1H), 7.51 (m, 3H), 7.62 (d, *J* = 8.2 Hz, 2H), 7.79 (m, 2H), 8.06 (dd, *J* = 7.8 Hz, *J* = 1.6 Hz, 1H), 8.92 (s, 1H), 9.61 (s, 1H). HRMS: *m*/*z* calcd for C_37_H_36_O_5_N_4_Cl_2_·H^+^ 687.21355; found 687.21174 (Δ = −1.81 ppm).

### 4.2. Cell Lines

DU145 (prostate carcinoma), A427 (lung carcinoma) and MDA-MB-468 (breast carcinoma) were obtained from the American Type Culture Collection.

HN (human oral-squamous-cancer cell lines derived from metastatic [23]) cells were obtained from the Leibniz Institute DSMZ (Braunschweig, Germany).

CAL33 and CAL166 cells (human head and neck squamous cell carcinoma, [24] and [25], respectively) were obtained from Centre Antoine-Lacassagne (Nice, France).

The 4T1 mouse mammary breast cancer cell line was obtained from Thierry Muanza’s Laboratory (McGill University, Montreal, Quebec, Canada). 

### 4.3. Cell Culture

All cell lines were maintained in an exponential growth phase in monolayer culture at 37 °C in a humidified atmosphere of 5% CO_2_ in a cell incubator. Cells were cultured in Dulbecco’s modified Eagle medium (DMEM)containing 10% fetal bovine serum (heat-decomplemented for HN cells) supplemented with 2 mmol/L L-glutamine and 4-(2-hydroxyethyl)-1-piperazine ethane sulfonic acid HEPES (10 mmol/L) for DU145 and A427.

### 4.4. In Vitro Drug Treatment

The combi-molecules AL530 and PD98059 were synthesized in the Cancer Drug Research Laboratory (McGill University Health Center). Chlorambucil (CBL) was purchased from Sigma–Aldrich. All drugs were dissolved in DMSO to obtain a solution at 5 mM for AL530 and PD98059 and 100 mM for CBL. Drug dilutions were carried out under sterile conditions in the culture medium where the DMSO concentration never exceeded 1% (*v*/*v*).

### 4.5. Growth Inhibition Assay and IC50 Determination

A total of 5000 cells/well for DU145, A427 and MDA-MB-468, 6250 for HN and 1800 for CAL33 and CAL166 were seeded in a 96-well plate in sextuplet in DMEM supplement as described above on day 0 (Section 4.3). After 24 h, cells were treated for four days with various concentrations of AL530, CBL or PD98059.

The number of cells was then determined by the sulforhodamine B assay as described by Skehan et al. [26]. Briefly, sulforhodamine B is a colorimetric assay that determines the protein sample content, which is directly correlated with the number of cells. Optical density was read for each well at 540 nm using a Labsystems Muttishan Multisoft microplate reader and IC50 was determined using the Graphpad Prism software package. Results are expressed as means ± standard error of the mean (SEM) and were representative of at least three independent experiments performed in sextuplet.

### 4.6. Temperature and pH Course Study of AL530 Hydrolysis

Solutions of 25 µM of AL530 were used in this series of experiments. These solutions (600 µL) were prepared using a Tris–base buffer (50 mM) at a pH ranging from 7 to 5.5. Each solution contained a maximum of 0.5% DMSO (*v*/*v*) incubated at 37, 40 or 42 °C for 96 h to study AL530 cleavage.

Every 24 h, 100 µL of each solution were collected from each incubated sample and then extracted for analysis by HPLC. After extraction, 20 µL of the organic phase were injected and compounds were eluted using an isocratic gradient of 60% acetonitrile and 40% H_2_O at 0.8 mL/min on a Waters LC–UV system. Results are expressed as means ± standard error of the mean (SEM) and represent at least three independent experiments. The retention times of AL530 and PD98059 were, respectively, 10.5 and 5.1 min. The rates of hydrolysis of the AL530 in PD98059 were determined by measuring the areas of peaks observed in the HPLC chromatograms per time point as per each experimental condition.

### 4.7. In Vivo Drug Treatment

#### 4.7.1. (a) Syngeneic 4T1 Mouse Model for Tumor Distribution Analysis

Experiments in the mouse 4T1 models were performed in female Balb/c mice (Charles River LaboratoriesMontreal, Quebec, Canada) strictly in accordance with the protocol #4934 approved by the Facility Animal Care Committee (FACC), McGill University. Briefly, mice were implanted subcutaneously with 4T1 mammary tumor cells suspended in 200 μL of phosphate buffered saline (PBS). Mice with a tumor size greater than 500 mm^3^ were divided into groups of 3, including vehicle control (saline/cremophor/ethanol 75/12.5/12.5). AL530 was administered i.p. (100 mg/kg) and after 3 h post-administration, the mice were sacrificed and their tumors and plasma collected. Samples were processed for analysis as described in the HPLC sample preparation section.

#### 4.7.2. (b) Xenograft Model

Female Swiss athymic nude mice (Charles River, France), 4–5 weeks old, were housed in filter-capped cages kept in a sterile facility and maintained in accordance with the FELASA (Federation of European Laboratory Animal Science Associations) standards. Following a two-week quarantine, mice were included in the protocols. The Claudius Regaud Institute animal ethics committee’s approval was obtained for the animal model and the study protocols.

Xenografts were established by subcutaneous injection of 10 × 10^6^ cells into both mouse flanks. When the tumors reached an average volume of 200 mm^3^ (day 4 post-implantation), the mice were pooled and randomly assigned to control or treated groups. Mice of the treated groups were i.p. injected with the following different drugs and doses: AL530 = 50 or 25 mg/kg, CBL = 11 mg/kg, PD98059 = 9.7 mg/kg, equimolar combination of CBL + PD98059. The vehicle (saline with less than 0.1 DMSO) and drugs (in saline and less than 0.1 DMSO) were administered daily for 12 days. Two perpendicular diameters of tumors were daily measured by the same investigator and caliper square. Tumor volume was calculated according to the following formula: V (mm^3^) = d^2^ (mm^2^)·D(mm)/2, where d and D, are the smallest and the largest tumor diameters, respectively. In each group, the relative tumor volume was expressed as a V^t^/V^0^ ratio where V^t^ is the mean tumor volume on a given day during the treatment, and V^0^ is the mean tumor volume at the beginning of the treatment. The maximum body weight loss and/or tumor volume tolerated by the ethics committee and the protocol were, respectively, 20% of initial weight and 2000 mm^3^. When tumor volume reached 1000 mm^3^, mice were inspected twice daily and sacrificed before the occurrence of poor health manifestations. At the end of the experiment, following anesthetization, the mice were sacrificed by total blood collection via cardiac puncture using heparinized syringes and collection tubes. Then, the tumors in each flank of the mice were removed and one tumor was fixed in formalin for pathological and immunohistochemical analysis and the other was frozen at −80 °C for the pharmacokinetics studies.

### 4.8. Pharmacokinetics

Pharmacokinetic studies were carried out by determining AL530 concentrations in plasma and tumors of mice having received a single dose of AL530 (50 mg/kg, i.p. route). Three mice per time point were implanted in both flanks with CAL33 cells (10 × 10^6^ cells). After one week and when tumor size reached 1000 mm^3^, mice were treated with the vehicle or 50 mg/kg i.p. of AL530 and sacrificed. Plasma and tumor tissue were collected at 10 min, 30 min, 1 h, 1.5 h, 2 h, 2.5 h, 4 h, 8 h and 24 h. Blood was collected in heparinized tubes, and plasma was separated from blood samples after centrifugation at 1500× *g* for 10 min at 4 °C. Tissues were immediately frozen at −80 °C until analysis. The data obtained by LC–UV were analyzed with the software NONMEM to generate the pharmacokinetics parameters.

### 4.9. HPLC Sample Preparation

Plasma samples were precipitated with acetonitrile (two volumes of acetonitrile for one volume of plasma), vortexed (30 s), centrifuged at 4 °C (15,000× *g*, 20 min) and stored at −20 °C for two hours for decantation in two phases (organic/aqueous). The resulting supernatant (organic phase) was collected and filtered using 0.2 µm of Nylon membrane (Thermo Fisher Scientific, Waltham, MA, USA) and dried, using a SpeedVac^®^ Concentrator (Eppenforf, Westbury, NY, USA) at room temperature.

When frozen tumor tissue samples were thawed, they were immediately cut into small pieces with a scalpel, introduced into a 2 mL tube with steel balls and ground with a QIAGEN Tissue Lyser II system (3 min). This tissue homogenate was added to 250 µL of denaturing buffer (9 M urea, 2% CHAPS, 50 mM Tris-HCl, pH: 9), vigorously vortexed for 30 min at room temperature before adding two volumes of acetonitrile. The mixture underwent a new vortex cycle for 1 min, followed by an 8 min sonication in an ultrasonic cleaner bath and centrifuged (15,000× *g*, 20 min at 4 °C). The supernatant was collected and cleaned through an HPLC filter (0.2 µm of Nylon), and the tube was stored at −20 °C for two hours leading to the separation of the two phases. The upper acetonitrile phase was transferred into a new evaporation tube to be dried using a SpeedVac^®^ Concentrator.

Prior to HPLC analysis, the dried samples were resuspended in 100 µL of acetonitrile, sonicated for 8 min in an ultrasonic cleaner bath and centrifuged at 4 °C (15,000× *g*, 20 min) before being injected for LC–UV analysis as described above (UV detection at 254 nm). Results are representative of at least two independent experiments.

### 4.10. Western Blot Analysis

On day 1, CAL33 cells (2 × 10^6^) were plated in the culture medium in 100 mm culture dishes. On day 2, for total and phosphorylated Erk1,2 analysis, cells were treated with either the vehicle or with AL530 at a concentration of 25 μg/mL for 48 h. The medium and treatment were changed every 24 h and the cells were harvested and lysed in a RIPA lysis buffer (Tris 50 mM pH 8, NaCl 150 mM, 0.1% NP40, 5 mM MgCl_2_, 50 mM NaF, 2 mM PMSF, 10 mM DTT, 2 mM orthovanadate, 5 mg/mL sodium dexoxycholate, 6.4 mg/mL phosphatase substrate; Sigma 104^®^). A total of 70 μg of proteins of the cleared lysates were separated onto a 12.5% SDS-PAGE gel, blotted onto PVDF membranes (Amersham, Orsay, France) and incubated with specific antibodies. Detection was performed using peroxidase-conjugated secondary antibodies (Bio-Rad) and an enhanced chemiluminescence detection kit (Amersham Pharmacia Biotech). The blots were scanned and analyzed with a Molecular Dynamics densitometer and ImageQuant software. Results are representative of at least two independent experiments.

### 4.11. Immunohistochemistry

The immunohistochemistry analyses were performed by the Department of Anatomic Pathology at the Claudius Regaud Institute. Tumors were collected from mice in control and treated groups. Thick sections of 4 µm of formalin-fixed and paraffin-embedded tumors from CAL33 cell xenografts were prepared and stained with antibodies against γ-H2AX and pErk1,2. Mouse data represent two independent experiments. Anti-phospho γ-H2AX and pErk1,2 antibodies were obtained from Santa Cruz.

### 4.12. Statistical Analysis

Data are expressed as mean ± S.E.M. Student’s two-sided t-test or Mann–Whitney test was used to compare values. Differences were considered statistically significant at *p* < 0.05.

## 5. Conclusions

This study demonstrated the feasibility of a complex combi-molecule designed to mask a kinase inhibitor and to release it in a pH range similar to the tumor microenvironment. We also demonstrated that the pharmacokinetics and distribution of the combi-molecule was markedly favorable to higher tumor than plasma concentration of its hydrolytic metabolites. Thus, as depicted by the model proposed in Figure 4B, the absorption and distribution of AL530 can be described as a partial hydrolysis in plasma and a more abundant conversion to the MEK inhibitor intratumorally. This apparent tumor selectivity may be due to an accelerated rate of cleavage favored by the more acidic pH of the tumor microenvironment when compared with plasma. Evidence of its ability to modulate its two primary targets (MEK and DNA), delay tumor growth and to induce less toxicity than CBL, a classical aniline mustard, suggests that this new approach warrants further investigation.

## Figures and Tables

**Table 1:** In vitro IC50 determination of AL530 in a panel of cancer cell lines.

Cell line	Tumor Type	AL530 IC50 (µM)
**DU145**	prostate	16.83 ± 1.10
**MDA-MB-468**	breast	3.15
**A427**	lung	3.33 ± 0.07
**HN**	head and neck	4.14 ± 0.18
**CAL166**	head and neck	20.31 ± 1.37
**CAL33**	head and neck	11.50 ± 1.32

**Table 2:** Growth inhibition in HN, CAL166 and CAL33 cell lines treated with different doses of AL530, CBL, PD98059 and combinations of CBL plus PD98059.

Drug	HN (µM)	CAL166 (µM)	CAL33 (µM)
**CBL**	21.34 ± 5.18	61.00 ± 8.92	57.4 ± 2.30
**PD98059**	nd	nd	nd
**CBL + fixed PD (20 µM)**	4.63 ± 0.70	nd	33.7 ± 4.40
**CBL + PD (1:1)**	12.3 ± 0.30	nd	24.0 ± 2.90
**AL530**	4.14 ± 0.18	20.31 ± 1.37	11.50 ± 1.32

**Table:** 

(A)		AUC_0–24h_ mg/µL·h	AUC_0–24h_ (mg/g·h)	T_1/2_ (h)	K_e_ (min^−1^)	C_max_ mg/µL for Plasma C_max_ mg/g for Tumor	t_max (h)_
**Plasma (P)**	**AL530**	3984.8	3,984,750	1.6	7.17	51.02	2
	**PD98059 released**	339.8	339,816.7	11.71	0.0009	3.61	2
**Tumor (T)**	**AL530**		6,393,616.7	3.93	2.94	32,345.62	2.5
	**PD98059 released**		1,658,845	6.77	0.0017	7054.31	2.5

**Table:** 

(B)	Ratio AUC_0–24h__Tumor/AUC_0–24h__Plasma
**AL530**	1.6
**PD98059**	4.88

## Data Availability

The data supporting this study are available in the article and its corresponding Supplementary Material section.

## Supplementary Materials

The following are available online at https://www.mdpi.com/1424-8247/14/2/160/s1, Scheme S1: (i) POCl_3_, pyridine, 0 °C to rt, 16 h, 83%, (ii) tBuOK, THF, rt, 16 h, 91%, (iii) H_2_SO_4_, rt, 1 h, 83%, (iv) Fe, AcOH, EtOH, H_2_O, reflux, 16 h, 59%, Scheme S2: (i) (1) SOCl_2_, DMF, reflux, 1 h, (2) TMSCHN2, THF/acetonitrile, Et_3_N, 0 °C, 1 h, filtration and evaporation, (3) Ag_2_O, MeOH, reflux, 6 h, (ii) MeI, NaH, 18-crown-6, DMF, 0 °C to rt, 6 h, 48% (step i + ii), (iii) NaOH 2N, MeOH, reflux, 3 h, 77%, (iv) MeOH, H_2_SO_4_, rt, 16 h, 94%, (v) SOCl_2_, reflux, Scheme S3: (i) Diethanolamine, DMF, 140 °C, 3.5 h, 57%, (ii) MsCl, Et_3_N, THF CH_2_Cl_2_, 0 °C, 1 h, 96%, (iii) LiCl, DMF, 110 °C, 30 min, 95%, (iv) SnCl_2_·2H_2_O, HCl, 100 °C, 7 h, 88%, Figure S1: NMR spectrum of PD98059 – 300 MHz, Figure S2: NMR spectrum of compound 7S – 300 MHz, Figure S3: NMR spectrum of compound 12S – 300 MHz, Figure S4: NMR spectrum AL530- 400 MHz, Figure S5: High Resolution Mass Spectrometry (HRMS) spectrum of AL530 - Positive Mode.

## Figures, Schemes and Tables

Table 3 (**A**) Pharmacokinetic parameters of AL530 and the released MEK inhibitor PD98059 and (**B**) the release ratio of PD98059 and AL530 in-between tumor and plasma.
