# Supplementary material for: Design and Mechanism of Action of a New Prototype of Combi-Molecule “Programed” to Release Bioactive Species at a pH Range Akin to That of the Tumor Microenvironment
Source: Pharmaceuticals (Basel). 2021 Feb 16;14(2):160. doi: 10.3390/ph14020160 (PMC7920489; doi:10.3390/ph14020160)
Supplement: Supplementary file 1 [file pharmaceuticals-14-00160-s001.pdf]

SUPPORTING INFORMATION

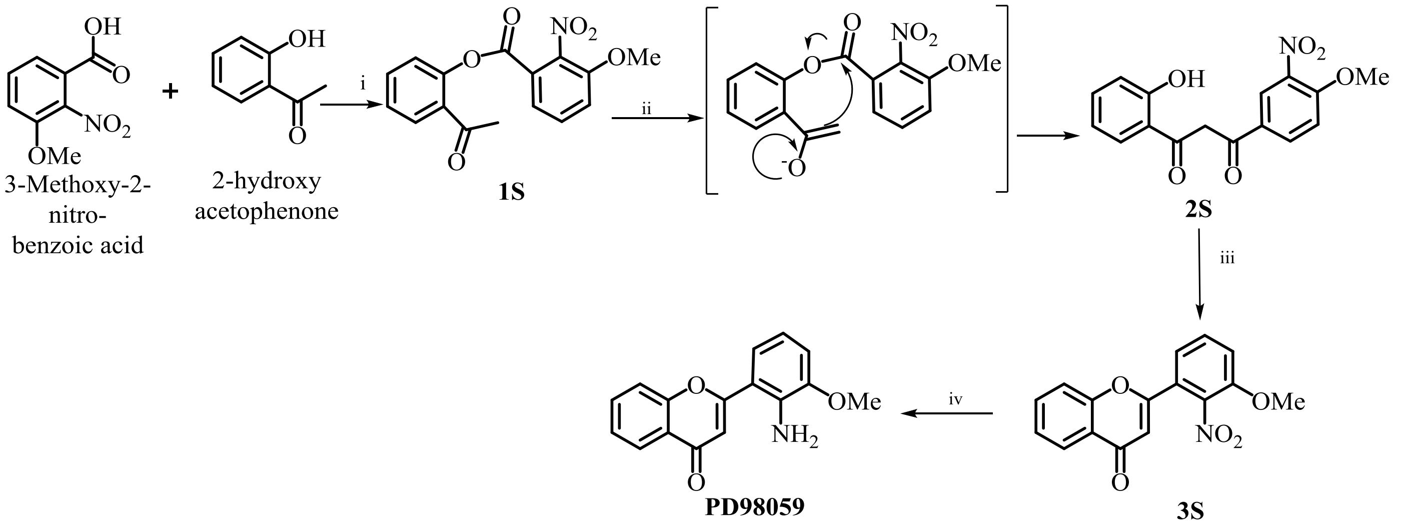

Scheme 1S. i) $POCl_3$, pyridine, 0°C to rt, 16h, 83%, ii) tBuOK, THF, rt, 16h, 91%, iii) H_2SO_4, rt, 1h, 83%, iv) Fe, AcOH, EtOH, H_2O, reflux, 16h, 59%.

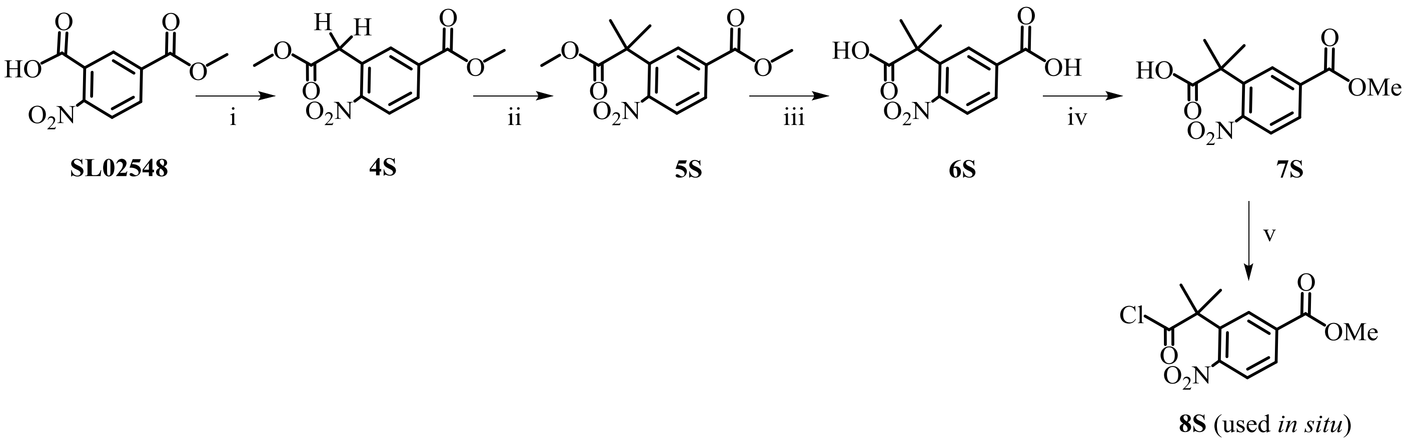

Scheme 2S. i) (1) $SOCl_2$, DMF, reflux, 1h, (2) $TMSCHN_2$, THF/acetonitrile, Et_3N, 0°C, 1h, filtration and evaporation, (3) Ag_2O, MeOH, reflux, 6h, ii) MeI, NaH, 18-crown-6, DMF, 0°C to rt, 6h, 48% (step i+ii), iii) NaOH 2N, MeOH, reflux, 3h, 77%, iv) MeOH, H_2SO_4, rt, 16h, 94%, v) $SOCl_2$, reflux.

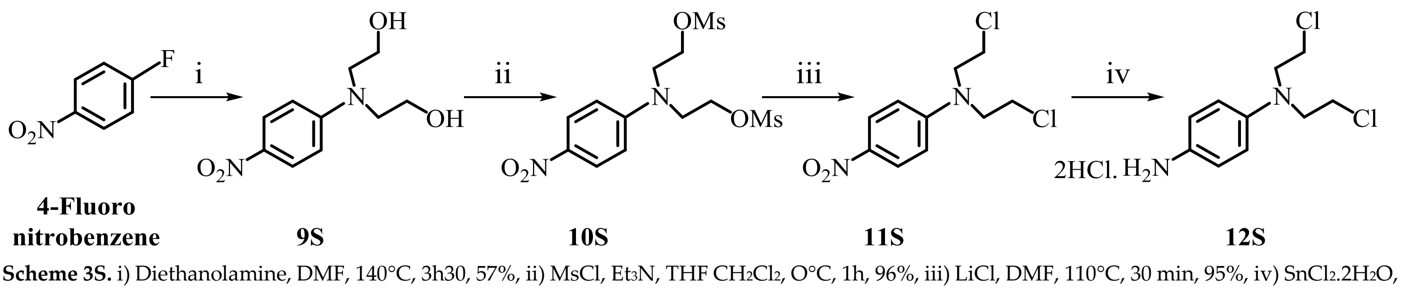

Scheme 3S. i) Diethanolamine, DMF, 140°C, 3h30, 57%, ii) MsCl, Et_3N, THF CH_2Cl_2, O°C, 1h, 96%, iii) LiCl, DMF, 110°C, 30 min, 95%, iv) $SnCl_2.2H_2O$, HCl, 100°C, 7h, 88%.

NMR spectrum of **PD98059** - 300MHz

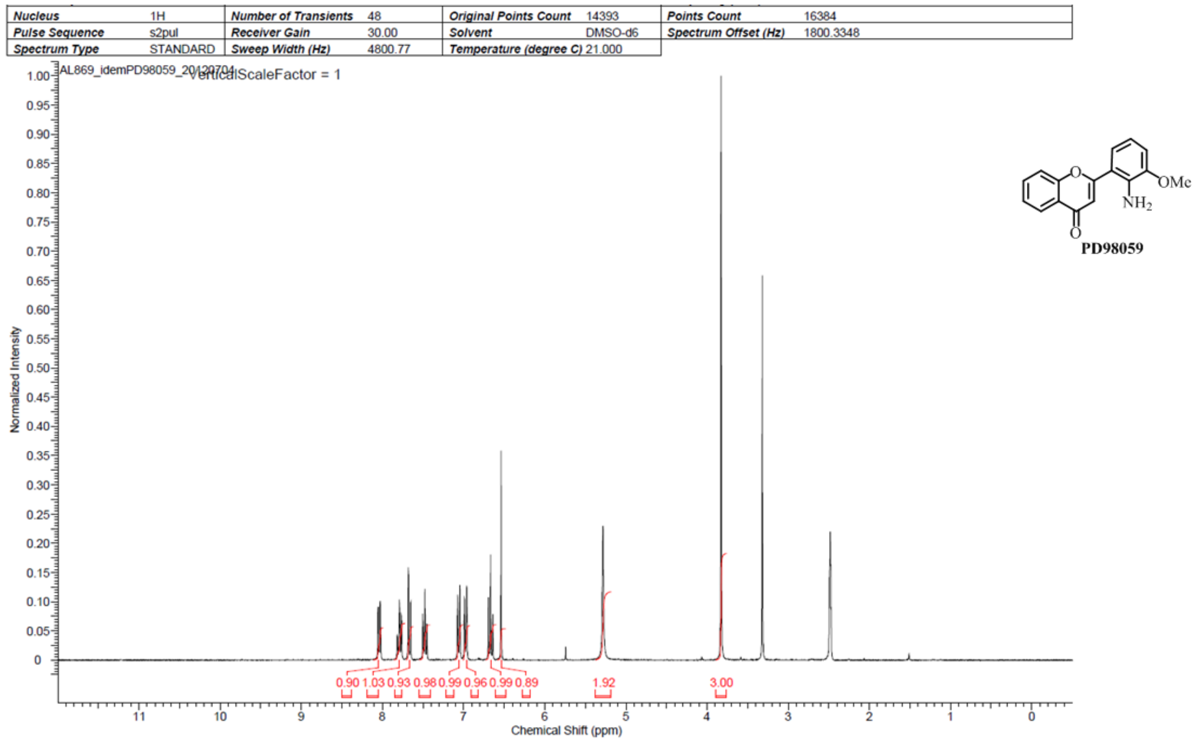

NMR spectrum of compound **7S** - 300MHz

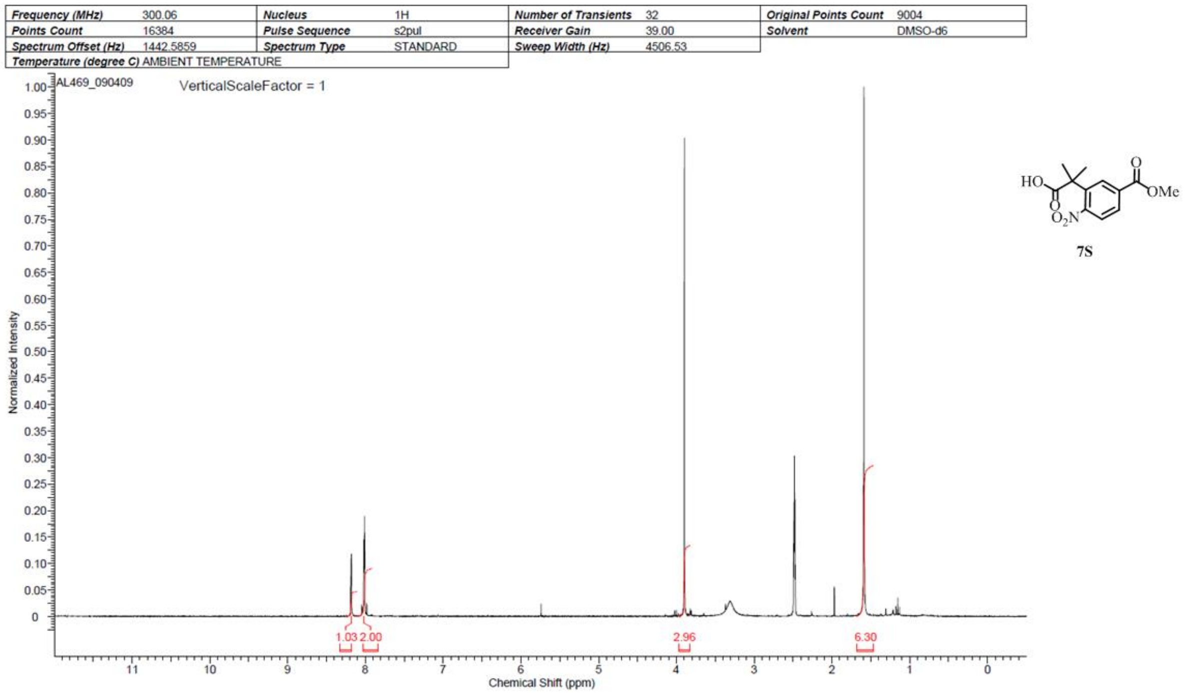

NMR spectrum of compound **12S** - 300MHz

Nucleus	1H	*Number of Transients*	16	*Original Points Count*	8992	*Points Count*	16384
Pulse Sequence	s2pul	*Receiver Gain*	32.00	*Solvent*	DMSO-d6	*Spectrum Offset (Hz)*	1442.5859
Spectrum Type	STANDARD	*Sweep Width (Hz)*	4506.53	*Temperature (degree C)*	AMBIENT TEMPERATURE		

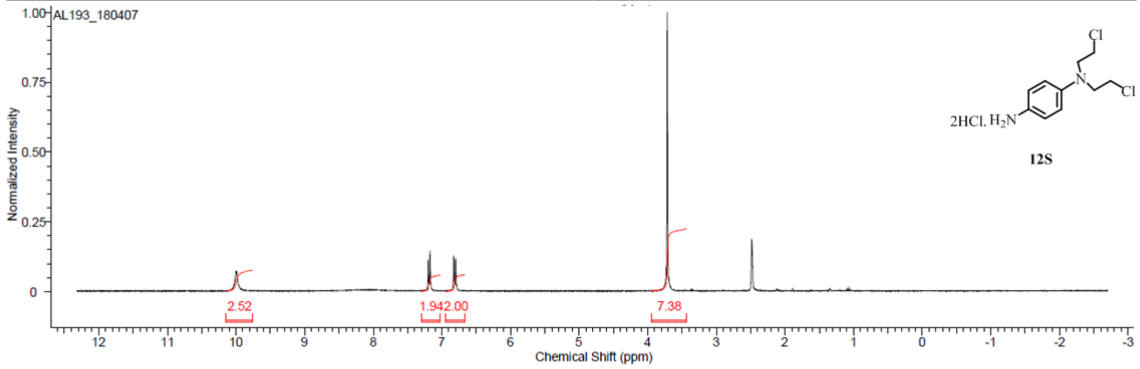

NMR spectrum **AL530**- 400MHz

Frequency (MHz)	400.13	*Nucleus*	1H	*Number of Transients*	48
Original Points Count	12788	*Points Count*	16384	*Pulse Sequence*	s2pul
Receiver Gain	16.00	*Solvent*	DMSO-d6	*Spectrum Offset (Hz)*	2407.5356
Spectrum Type	STANDARD	*Sweep Width (Hz)*	6410.26	*Temperature (degree C)*	AMBIENT TEMPERATURE

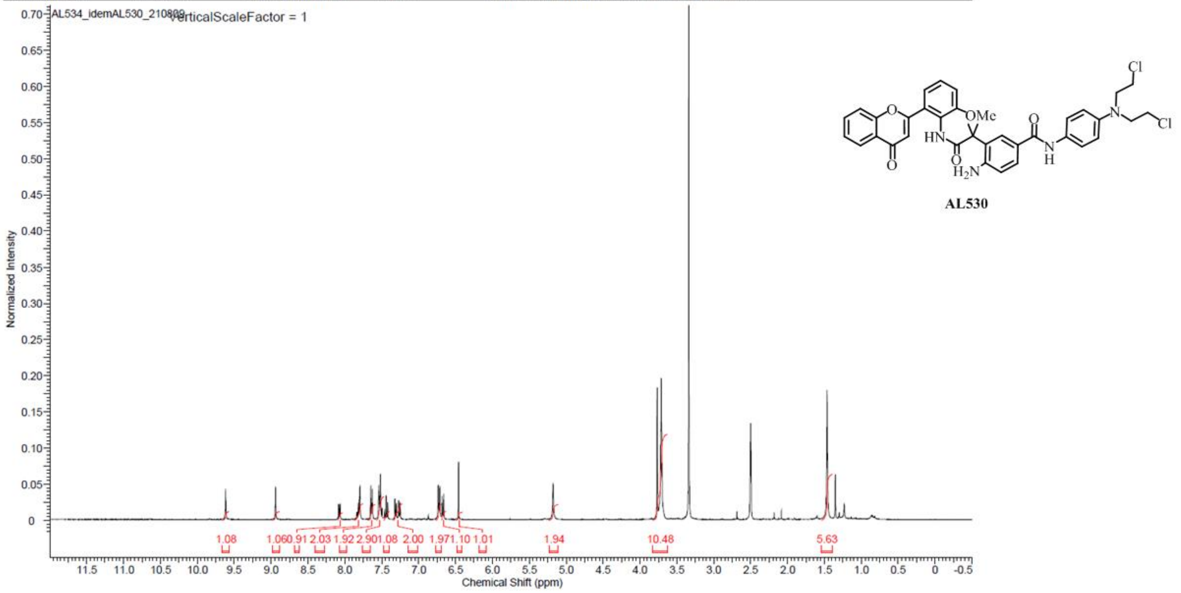

High Resolution Mass Spectrometry (HRMS) spectrum of **AL530** - Positive Mode

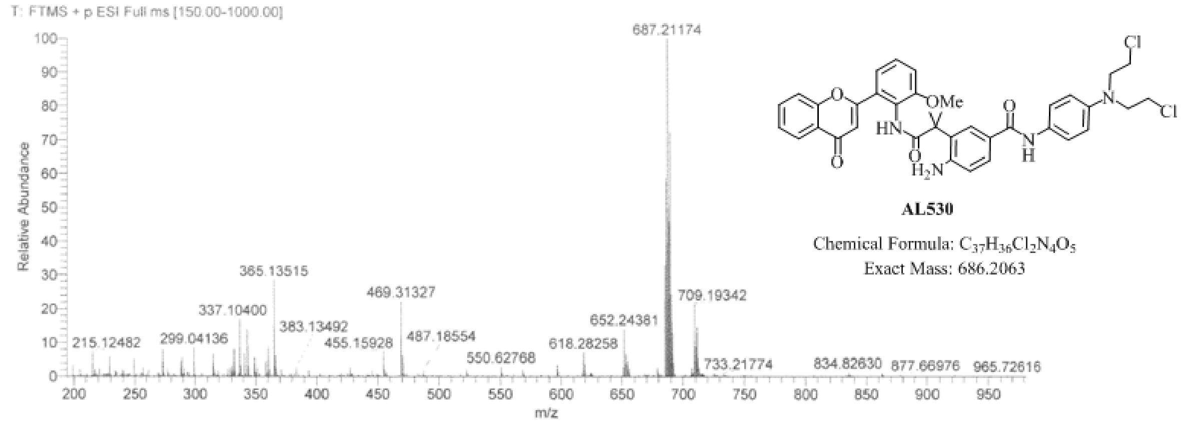

AL530

Chemical Formula: $C_{37}H_{36}Cl_2N_4O_5$

Exact Mass: 686.2063

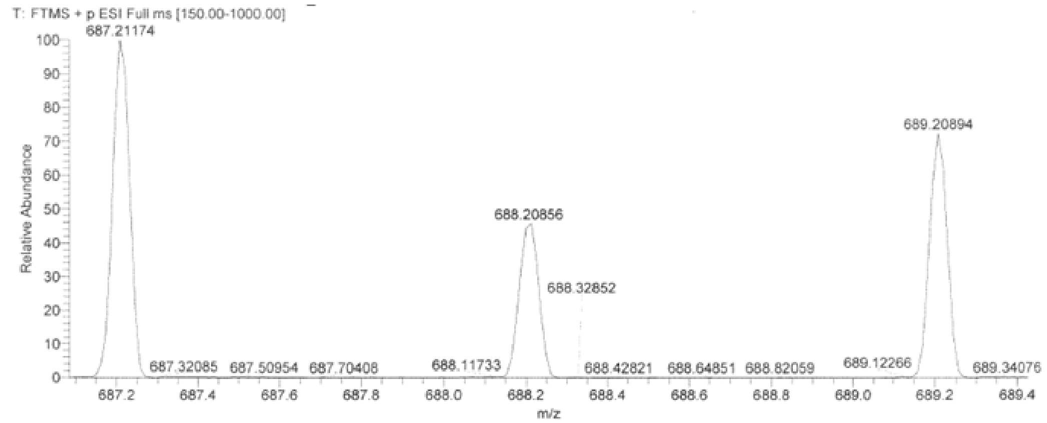

T: FTMS + p ESI Full ms [150.00-1000.00]
m/z= 687.15629-687.27405

m/z	Intensity	Relative	Resolution	Charge	Theo. Mass	Delta (ppm)	RDB equiv.	Composition
687.21174	287191.2	100.00	15039.25	1.00	687.21355	-1.81	20.5	$C_{37}H_{37}O_5N_4Cl_2$
					687.20902	2.72	29.0	$C_{46}H_{35}ONCl_2$
					687.20853	3.20	33.5	$C_{49}H_{32}O_2Cl$
					687.21526	-3.52	34.0	$C_{46}H_{29}O_4N_3$
					687.21575	-4.01	29.5	$C_{43}H_{32}O_3N_4Cl$
